# MicroRNAs coordinately regulate protein complexes

**DOI:** 10.1186/1752-0509-5-136

**Published:** 2011-08-25

**Authors:** Steffen Sass, Sabine Dietmann, Ulrike Burk, Simone Brabletz, Dominik Lutter, Andreas Kowarsch, Klaus F Mayer, Thomas Brabletz, Andreas Ruepp, Fabian Theis, Yu Wang

**Affiliations:** 1 MIPS, Institute for Bioinformatics and System Biology, Helmholtz Center Munich, German Research Center for Environmental Health, Ingolstädter Landstraße 1, D-85764 Neuherberg, Germany; 2 Wellcome Trust Center for Stem Cell Research, University of Cambridge, Tennis Court Road, Cambridge CB2 1QN, UK; 3 Department of Visceral Surgery, Universitätsklinikum Freiburg, Hugstetter Strasse 55, D-79106, Freiburg, Germany; 4 Center for Life and Food Sciences Weihenstephan, Technicial University Munich, Emil-Ramann-Str. 4, D-85354 Freising, Germany

## Abstract

**Background:**

In animals, microRNAs (miRNAs) regulate the protein synthesis of their target messenger RNAs (mRNAs) by either translational repression or deadenylation. miRNAs are frequently found to be co-expressed in different tissues and cell types, while some form polycistronic clusters on genomes. Interactions between targets of co-expressed miRNAs (including miRNA clusters) have not yet been systematically investigated.

**Results:**

Here we integrated information from predicted and experimentally verified miRNA targets to characterize protein complex networks regulated by human miRNAs. We found striking evidence that individual miRNAs or co-expressed miRNAs frequently target several components of protein complexes. We experimentally verified that the *miR-141-200c *cluster targets different components of the *CtBP/ZEB *complex, suggesting a potential orchestrated regulation in epithelial to mesenchymal transition.

**Conclusions:**

Our findings indicate a coordinate posttranscriptional regulation of protein complexes by miRNAs. These provide a sound basis for designing experiments to study miRNA function at a systems level.

## Background

Hundreds of microRNA (miRNA) genes have been identified in mammalian genomes [1]. Each miRNA may repress the translation of, and/or destabilize numerous messenger RNAs (mRNAs). Moreover, miRNA genes are frequently organized into genomic clusters [2-4], which are transcribed from a common promoter as polycistronic primary transcripts, and whose coordinate functional roles remain to be investigated [5]. Recent large-scale, quantitative proteomics studies have demonstrated that some miRNAs probably participate in fine-tuning the production of their targets, both at the messenger RNA and the protein level [6,7]. However, the overall effect of miRNAs on many of their target proteins is often intriguingly modest. It remains unclear how these marginal effects can convey the necessary regulatory information for proper cellular activities [8].

We applied a network-based strategy to systematically map coordinate regulatory interactions of single and co-expressed (including clustered) miRNAs. Previous works [9-12] have demonstrated that the targets of single miRNAs are more connected in the protein-protein interaction network than expected by chance. The use of protein-protein interaction (PPI) data provides only a rough overall picture of miRNA target interactions. It is not easy to evaluate the regulatory effects of miRNAs on such large-scaled PPI networks. Instead, as the basic functional units of the cellular machinery, experimentally verified protein complexes are natural subsets of PPI networks for investigating miRNA target interactions. Several components of protein complexes may be regulated simultaneously by a single miRNA or by several co-expressed miRNAs. Thus, although the regulation of protein synthesis is marginal for some of the miRNA targets, a cumulative effect for substantial phenotypic consequence may be achieved for those targets, which are members of the same protein complexes.

To test this hypothesis, we developed a robust computational framework to select protein complexes, of which several distinct components are simultaneously regulated by either single miRNAs or co-expressed miRNAs. We applied the framework to characterize the protein complex networks, which consist of 722 experimentally verified protein complexes and protein-protein interactions. These protein complex networks are regulated by 677 miRNAs and 154 known miRNA clusters in humans. We find that our framework has several advantages over previous analyses of miRNA targets and their interactions. First, high-confidence miRNA target predictions allowed us to characterize the overall functional spectrum of miRNA-regulated protein complexes. Second, we demonstrated that miRNAs, which target the same protein complexes, are frequently co-expressed. Finally, we experimentally verified that the *miR141-200c *cluster simultaneously targets several protein components of the CtBP/ZEB complex, implying an efficient regulation of a protein complex by a cluster of miRNAs.

## Methods

### miRNA targets and target interaction networks

Recent studies showed a high reliability of miRNA targets predicted by TargetScan [7]. Therefore we selected the targets for all human miRNAs listed in the TargetScan database. We obtained a set of 677 miRNAs and 18,880 unique target proteins. The resulting miRNA-protein network contained 224,316 interactions. To predict miRNA targets based on PAR-CLIP data, the crosslink-centered regions (CCRs) from combined AGO-PAR-CLIP libraries [13] were used. Target site prediction for all CCRs was done with the program RNAhybrid [14] with the default parameters. From the resulting list we filtered all predictions with a p-value below 0.02 and an energy score below the 25% quantlile. This resulted in a final miRNA- mRNA list of 50,160 predicted interactions.

### Association of protein complexes with miRNA target sets - test for statistical significance

We used the Fisher's exact test for assigning the significance of the association with protein complexes for each miRNA target set. The hypergeometric P-value is given as the probability under which we could expect at least *N_c _*miRNA targets by chance in a protein complex, if we randomly select *N_t _*(total number of miRNA targets) proteins out of the total set of proteins N consisting of all miRNA targets *N_T _*and all proteins in complexes *N_C _*. P-values were corrected for multiple testing of 677 miRNAs using the Holm-Bonferroni correction method. We assigned the association of complexes and miRNA clusters by using the union of targets from all miRNAs within one cluster. Here, we tested for significant overlaps of these unified sets between the components of a complex in the same way as for single miRNA target sets.

### Enrichment of biological processes

In order to test for significant enrichment of biological functions based on Gene Ontology (GO) [15] and KEGG [16] pathways within the set of targets in protein complexes, the R package GOstats [17] was used. A set of targeted components of 722 targeted protein complexes was extracted and compared to a set of proteins which consisted of all components of these complexes.

### Comparison of fold change distributions

We used fold change measurements after over-expression of selected miRNAs from recent proteomics studies [6,7]. We selected for every of these miRNAs the protein complexes consisting of at least one of its targets. A set of components of these protein complexes was built. Within this set, we compared the fold changes of components that are targets of the specific miRNA with the fold changes of the non-target components. This was done by performing a one sided Kolmogorov-Smirnov test for each of the miRNAs that were investigated in the proteomics studies.

### Cell culture

PANC-1 cells were purchased from ATCC (Manassas, VA, USA). PANC-1 stable clones for *miR-141 *or *miR-200c *were obtained with sequence verified pRetroSuper-miRNA plasmids. Cell lines were cultivated under standard conditions in DMEM + 10% fetal bovine serum + 2 μg/ml puromycin. For transient knock down PANC-1 were transfected with siRNA targeting ZEB1 (r(aga uga uga aug cga guc g)d(TT)), CtBP2 (1: r(cuuuggauucagcgucaua)d(TT), 2: r(cuuuguaacugauucugga)d(TT)) or GFP (r(gcu acc ugu ucc aug gcc a)d(TT). All transfections and reporter assays were performed as described previously [18].

### Specific assay for miRNA modulation

RNA from cultured cells was extracted using the mirVana™ miRNA Isolation Kit (Ambion, Austin, TX, USA). mRNA expression values were measured in triplicate using the Roche LightCycler 480 and normalized to b-actin expression as a housekeeping control. Expression values were calculated according to ref.[19].

### Immunoblots

were performed using modified standard protocols. In brief, whole cell extracts were made of the cells in Triple Lysis Buffer [50 mM Tris-HCl pH8, 150 mM NaCl, 0,02% (w/v) NaN_3_, 0,5% (w/v) NaDeoxycholate, 0,1% SDS, 1% (v/v) NP40]. Extracts (10 μg/lane) were separated on a 10% SDS-polyacrylamide gel, blotted onto a PVDF membrane, and incubated with the indicated primary antibodies diluted in blocking buffer (5% nonfat dry milk) over night at 4°C. After washing and incubation with peroxidase-coupled species-specific secondary antibodies, the signal was developed using SuperSignal West PICO Chemiluminescent Substrate (Perbio Science, Bonn, Germany) according to manufacturer's protocol. *CtBP2, CDYL, RCOR3, β-actin *and *ZEB1 *were immunodetected with the following primary antibodies: anti-*CtBP2 *mouse monoclonal antibody (1:8.000, BD Transduction Laboratories™, Franklin Lakes, NJ, USA), anti-*CDYL *rabbit polyclonal antibody (1:500, Abcam, Cambridge, UK), anti-*RCOR3 *rabbit polyclonal antibody (1:1000Abcam, Cambridge, UK) anti-*β-actin *mouse monoclonal antibody (1:5.000, Sigma-Aldrich Chemie GmbH, Munich, Germany). The anti-*ZEB1 *rabbit polyclonal antibody (1:20.000) was a gift of D.S. Darling, University of Louisville, Louisville, KY, USA.

## Results

In order to identify protein complexes of which several distinct components are coordinately regulated by miRNAs, we assembled a miRNA-protein target network for 677 human miRNAs and 18,880 targets which are listed in the TargetScan http://www.targetscan.org database. The targets were mapped to a non-redundant set of 2,177 experimentally verified protein complexes from the CORUM database [20]. We compiled the protein complexes, which are more significantly associated with the target sets of miRNAs than expected for random target lists based on Fisher's exact test (see Methods). The analysis resulted in 722 miRNA-regulated protein complexes (P-value < 0.05; Fisher's exact test with Bonferroni correction for multiple testing), which contained at least two targets of an individual miRNA. The entire list of miRNA-regulated protein complexes can be found in Additional file 1, Table S1 online. Furthermore, 140 protein complexes were significantly regulated by miRNA clusters (P-value < 0.05, Fisher's exact test with Bonferroni correction for multiple testing). The list of protein complexes regulated by clusters of miRNA can be found in Additional file 2, Table S2. The highest ranked complexes are listed in Table 1 and Table 2.

**Table 1:** Top ranking single miRNAs targeting protein complexes

Complex	Description	miRNA	P-value
corum_3028	TGF-beta receptor II-TGF-beta receptor I-TGF-beta3 complex	hsa-miR-665	2.00326E-05
corum_1810	ITGA4-PXN-GIT1 complex	hsa-miR-199a-5p	3.26913E-05
corum_4	ACTR-p300-PCAF complex	hsa-miR-338-5p	3.65869E-05
corum_642	CtBP complex	hsa-miR-129-5p	4.60618E-05
corum_642	CtBP complex	hsa-miR-548f	5.10388E-05
corum_3754	CREBBP-SMAD3-SMAD4 pentameric complex	hsa-miR-1284	7.21639E-05
corum_3753	CREBBP-SMAD2-SMAD4 pentameric complex	hsa-miR-1264	8.26908E-05
corum_2377	ITGA2b-ITGB3-CD47-SRC complex	hsa-miR-149	8.78087E-05
corum_2760	SMAD3-SMAD4-FOXO3 complex	hsa-miR-1284	9.18449E-05

**Table 2:** Top ranking miRNA clusters targeting protein complexes

Complex	Description	Cluster	P-value
corum_3028	TGF-beta receptor II-TGF-beta receptor I-TGF-beta3 complex	hsa-miR-493-665	0.00079949
corum_3753	CREBBP-SMAD2-SMAD4 pentameric complex	hsa-miR-1912-1264	0.00095073
corum_3059	ITGA11-ITGB1-COL1A1 complex	hsa-miR-29a-29b	0.00101944
corum_3059	ITGA11-ITGB1-COL1A1 complex	hsa-miR-29c-29b	0.00101944
corum_1080	P-TEFb.2 complex	hsa-miR-224-452	0.00168046
corum_3054	MAD1-mSin3A-HDAC2 complex	hsa-miR-1912-1264	0.00316828
corum_3054	MAD1-mSin3A-HDAC2 complex	hsa-miR-510-514	0.00316828
corum_422	Beta-dystroglycan-caveolin-3 complex	hsa-miR-3671-101	0.00330472
corum_2436	ITGAV-ITGB1 complex	hsa-miR-513c-513b	0.00333788

### Functional spectrum of miRNA-regulated protein complexes

**Figure 1:**
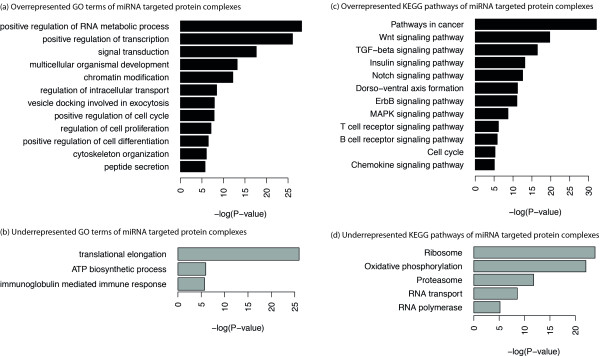
**Functional analysis and validation of miRNA-regulated protein complexes**. Functional analysis: Enrichment of Gene Ontology (GO) terms and KEGG pathways in the target subunits of protein complexes. The size of the bars for each term indicates the negative logarithm of the P-value. Only meaningful and non-redundant terms were selected for illustration. See Additional file 3 &4, Table S3&S4 for a complete and detailed list of significant terms.

We next analyzed the spectrum of functions covered by our set of miRNA-regulated protein complexes. We identified the biological processes (Gene ontology categories [15]) and pathways representing the molecular interactions and reaction networks (KEGG [16]), which are enriched within the total set of 810 miRNA-targeted components of the protein complexes (Additional file 3, Table S3 and Additional file 4, Table S4 online). In all, as shown in Figure 1a, the miRNA-regulated protein complexes are mainly involved in regulation of RNA metabolic process, regulation of transcription and chromatin modification. Conversely, house-keeping functions, such as translational elongation and ATP synthesis coupled electron transport are underrepresented. The results confirm earlier investigations [21] showing that miRNAs less frequently target genes involved in essential cellular processes. Interestingly, there is an overrepresentation of genes involved in the G1 phase of mitotic cell cycle, while genes that are involved in the S phase and the M/G1 transition of mitotic cell cycle are underrepresented. Experimental evidence has already been reported for the regulation of signal transduction in several metazoan species [22-26] and the cell cycle [27,28] by miRNAs. The regulation of the cell cycle by miRNAs is further supported by strong correlations of miRNA over-expression with different types of cancer [29].

These observations correspond with the overrepresentation of targeted genes contained in pathways from KEGG (see Figure 1b). A high overrepresentation of genes could be observed in "Pathways in cancer". Also many signaling pathways are overrepresented, namely Wnt signaling, TGF-beta signaling, Insulin signaling, Notch signaling, ErbB signaling, MAPK signaling, T and B cell receptor signaling and Chemokine signaling. Genes involved in house-keeping functions were underrepresented also in KEGG pathways, namely RNA polymerase, RNA transport, Proteasome, Oxidative phosphorylation and Ribosome.

#### Validating predicted miRNA targets in protein complexes

**Figure 2:**
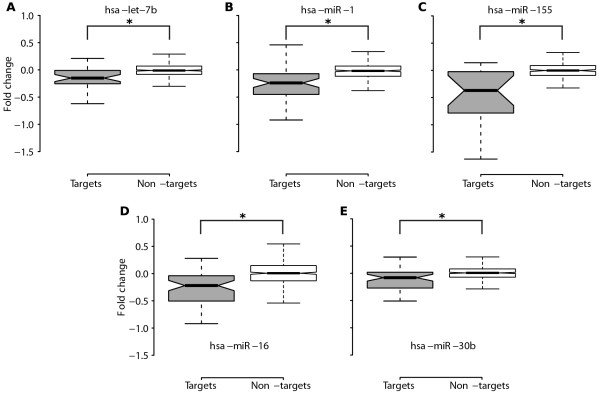
**Validation of targeted complex components**. Fold change distributions of targeted and non-targeted proteins in complexes for each investigated miRNA. The (*) indicates high significance in the Kolmogorov-Smirnov test.

Two recent proteomics studies measured the changes in synthesis of proteins in response to miRNA over-expression or knockdown on a genome-wide scale for selected miRNAs [6,7]. We incorporated the data of these studies in order to validate our predictions. To determine the impact of protein downregulation by miRNAs, which have targets in protein complexes, the level of downregulation of targeted components and non-targeted components was compared. We considered both significantly and insignificantly regulated complexes, since the amount of significantly regulated complexes for the examined miRNAs in the proteomics study is too low to provide statistical significance. The negative fold changes of the targeted components were significantly higher than the negative fold changes of the non-targeted components (see Table 3 and Figure 2) for every analyzed miRNA. For example, our data showed that the LARC (LCR-associated remodelling) complex [30] has two (out of 19) components, which are computationally predicted targets of let-7. These two components, namely DPF2 (Zinc finger protein ubi-d4) and SMARCC1 (SWI/SNF-related matrix-associated actin-dependent regulator of chromatin subfamily C member 1) were modestly down-regulated (fold changes of -0.38, and -0.2, respectively), when let-7b was over-expressed in HeLa cells [7]. LARC binds to the DNase hypersensitive 2 site in the human β-globin locus control region (LCR) and transactivates β-like globin genes [30]. By simultaneously down-regulating two components of the LARC complex, let-7b might contribute to the overall transcriptional repression of the human β-globin locus.

**Table 3:** Significance of miRNA target downregulation

miRNA	P-value
hsa-let-7b	1,5E-05
hsa-miR-1	5,1E-12
hsa-miR-155	5,3E-04
hsa-miR-16	1,8E-08
hsa-miR-30b	1,6E-03

The P-value was calculated by a one-sided Kolmogorov-Smirnov test. It was used to compare the fold change distributions of complex components that are miRNA targets and non-target ones.

PAR-CLIP (Photoactivatable-Ribonucleoside-Enhanced Crosslinking and Immuno-precipitation) is a powerful tool to detect segments of RNA bound by RNA-binding proteins (RBPs) and ribonucleoprotein complexes (RNPs). We corroborated the miRNA target sites identified by PAR-CLIP [13] with the proteomics data [6,7]. 55% of the proteins with miRNA targets sites predicted based on PAR-CLIP data were moderately down-regulated (log2-fold change < -0.1). 413 protein complexes contained miRNA target sites in at least two subunits (Additional file 5, Table S5 online). Interestingly, of the 5,185 unique proteins with miRNA target sites identified based on PAR-CLIP data, 607 (12%) are members of protein complexes (with at least two distinct targets of one miRNA in the same protein complex). For comparison, the manually curated collection of human protein complexes in the CORUM database covers 2,780 unique proteins (2% of UniProt proteins). This implies miRNA targets identified from PAR-CLIP data are more likely to be in a protein complex from the CORUM database (12%) as compared to proteins in general (2%). While miRNAs frequently target multiple genes with isolated functions, these independent data, though only by a simple estimate, suggest that there is also a significant proportion of miRNA targets, which are distinct members of protein complexes (hypergeometic P-value 1.23e-11).

### Protein complexes and miRNA expression

We next tested whether miRNAs, which target different components of the same protein complex, are more likely to be co-expressed. The average expression correlation (Co-expression as calculated by Pearson correlation coefficients, hereafter termed PC values) of miRNAs was examined based on pairwise correlation calculations of miRNA expression profiles obtained for 26 different organ systems and cell types [31]. To test for statistical significance, we combined all pairwise PC values obtained from the sets of miRNAs which significantly target the same complex. These PC values were then compared to all other pairwise PC values that were present in the data set from [31]. We performed a one-sided Kolmogorov-Smirnov (KS) test for the two PC value distributions and obtained a significantly (P-value 6.106e-24) higher co-expression within the sets of miRNAs that target the same complex. Since we are interested in coexpression of miRNAs that are not in one transcription unit, we also tested for increased correlation only for miRNAs of different transcription units. Only a few (3.3%) of the correlated miRNAs were actually contained in one transcription unit. Therefore, the result remains highly significant (P-value 2.11e-18). Another bias of our results might occur due to fact that all miRNAs from one family must target the same complex since they target the same set of mRNA. We compared only miRNAs within one complex that belong to different families. The KS test resulted in a P-value of 0.0058. Taken together, our statistical test indicates that miRNAs targeting different components of a protein complex are significantly co-expressed. The average Pearson correlations of miRNAs that simultaneously target a specific complex can be found in Additional file 6, Table S6 online1).

### Protein complex networks co-ordinately regulated by clusters of miRNAs

**Figure 3:**
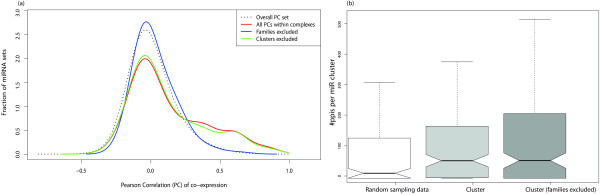
**Statistical evidence of coordinate regulation by miRNAs**. **a**, Pearson correlation distributions of miRNAs that target the same complex (red line) is plotted against the distribution of all observed Pearson correlation values (black dotted line). Also the distributions of excluded Pearson correlations of miRNAs from the same family (blue) and the same cluster (green) are plotted. **b**, Boxplot for direct interactions of proteins targeted by *N *miRNAs within a cluster as compared to a null model of *N *randomly sampled miRNAs, respectively.

We systematically characterized the protein complex networks, which are simultaneously regulated by clustered miRNAs in 154 transcription units gained from miRBase [1]. The interconnectivity of the target sets of the miRNA gene clusters was first assessed as follows: the number of protein-protein interactions between the target sets of each pair of miRNAs in the cluster was counted, and these values were compared to 1,000 randomly sampled sets of miRNAs. To avoid miRNA target prediction bias arising from redundant prediction of clustered miRNA family members, only targets of one family member were counted within each cluster. The statistical analysis revealed 35 clusters, whose targets are significantly interconnected in the protein-protein interaction network (P-value < 0.05, permutation test, 1,000 samples, Table 1). Comparing the observed number of interactions (Figure 3b) with the corresponding distributions of randomly sampled sets of miRNAs provides a strong indication that a significant fraction of miRNAs in clusters might co-ordinately regulate targets (P-Value < 0.02, Wilcoxon signed rank test, Additional file 7, Table S7 online). In order to support this finding, we also applied Fisher's exact test to test if the global number of target interactions from miRNA clusters is higher than expected by chance. This test resulted in a P-value < 2e-16.

### CtBP/ZEB complex regulated by the miR-141-200c cluster

The network perspective provides fascinating insights of gene regulation by miRNA gene clusters, whose target sets have not yet been analyzed at a systems-level. To explore this in detail we examined the protein complexes predicted to be co-ordinately regulated by the *miR-141-200c *cluster. The *miR-141 *and *miR-200c *genes are located on chromosome 12p 13.31, separated by a 338bp spacer sequence; *miR-141 *and *miR-200c *belong to the *miR-200 *family. The seed region of *miR-141 *differs to that of *miR-200c *by one nucleotide at position 4 of the miRNA; therefore, *miR-141 *and *miR-200c *have, based on the "seed" rule, different computationally predicted targets. Nevertheless, we found that the targets of the *miR-141-200c *cluster are significantly interconnected (P-value < 0.02, Table 4).

**Table 4:** Top ranking miRNA clusters with interconnected target sets

miRNA cluster^15,^*	# targ	Ppis [#| P-value]		Ppis [miR-miR] [# |P-value]	
*hsa-miR-3671-101*	116	94	0.1294	94	0

*hsa-let-7a-7b*	120	48	0.1748	48	0

*hsa-miR-1912-1264*	49	24	0.3224	24	0

*hsa-miR-214-199a*	69	22	0.3222	22	0

*hsa-miR-296-298*	52	24	0.3324	24	0

*hsa-miR-105-767*	58	18	0.3418	18	0

*hsa-miR-34b-34c*	43	22	0.3522	22	0

*hsa-miR-3677-940*	55	18	0.3618	18	0

*hsa-miR-1914-647*	38	20	0.3720	20	0

*hsa-miR-599-875*	23	6	0.536	6	0

*hsa-miR-16-15a*	172	172	0.12172	172	0

*hsa-miR-15b-16*	172	172	0.15172	172	0

*hsa-miR-181c-181d*	160	142	0.21142	142	0

*hsa-miR-195-497*	172	172	0.15172	172	0

*hsa-miR-181a-181b*	160	142	0.19142	142	0

*hsa-miR-181b-181a*	160	142	0.18142	142	0.01

*hsa-miR-30e-30c*	183	144	0.2144	144	0.01

*hsa-miR-513c-513b*	125	142	0.21142	142	0.01

*hsa-miR-23b-24*	305	584	0.02234	234	0.01

*hsa-miR-363-106a*	341	550	0.29823	823	0.01

*hsa-miR-200c-141*	246	392	0.02112	112	0.01

*hsa-miR-30b-30d*	183	144	0.2144	144	0.01

*hsa-miR-519a-1283*	298	702	0.02258	258	0.01

*hsa-miR-24-23a*	305	584	0.03234	234	0.02

*hsa-miR-522-1283*	323	864	0.02377	377	0.02

*hsa-miR-25-106b*	233	236	0.22224	224	0.02

*hsa-miR-301b-130b*	123	86	0.3486	86	0.03

*hsa-miR-182-183*	218	288	0.15196	196	0.03

*hsa-miR-17-92a*	341	550	0.28607	607	0.03

*hsa-miR-133a-1*	189	262	0.0574	74	0.03

*hsa-miR-545-374a*	180	212	0.0862	62	0.04

*hsa-miR-206-133b*	189	262	0.0774	74	0.04

*hsa-miR-29a-29b*	126	64	0.3864	64	0.05

*hsa-miR-513a-508*	242	256	0.0559	59	0.05

*hsa-miR-513a-507*	242	256	0.0559	59	0.05

*miRNA cluster is termed in the following way: *miR-first_miRNA-last_miRNA*. For instance, *miR-17-92a *cluster is consisted of six miRNAs, *miR-17 *is the first miRNA in the cluster and *miR-92a *is the last miRNA in the cluster.

Interconnectivity of the target sets was evaluated as: (1) Number of interactions in the union target set and (2) Number of interactions between target sets of all distinct miRNA pairs in a cluster (ppis [miR-miR]). The P-values were estimated by comparing the observed value with 1,000 randomly sampled target sets of equal size. The results for all clusters are shown in Additional file 7, Table S7 online.

Very recent reports have shown that the *miR-200 *family regulates epithelial to mesenchymal transition (EMT) by targeting the transcriptional repressor zinc-finger E-box binding homebox 1 (*ZEB1*) and *ZEB2 *[4,32-35]. During EMT, the *miR-141-200c *cluster and the tumor invasion suppressor gene *E-cadherin *are downregulated by *ZEB1/2 *[35]. ZEB1 and ZEB2 repress transcription through interaction with corepressor CtBP (C-terminal binding protein) [36]. Interestingly, several essential components of the CtBP/ZEB complex, namely ZEB1/2, CtBP2, RCOR3 (REST corepressor 3) and CDYL (Chromodomain Y-like protein), are predicted targets of the miR-141-200c cluster. CtBP2 has one *miR-141 target site *and one miR-200c target site, while ZEB1 and CDYL have two miR-200c target sites. RCOR3 has one miR-141 target site. The CtBP/ZEB complex mediates the transcriptional repression of its target genes by binding to their promotors and altering the histone modification [37].

**Figure 4:**
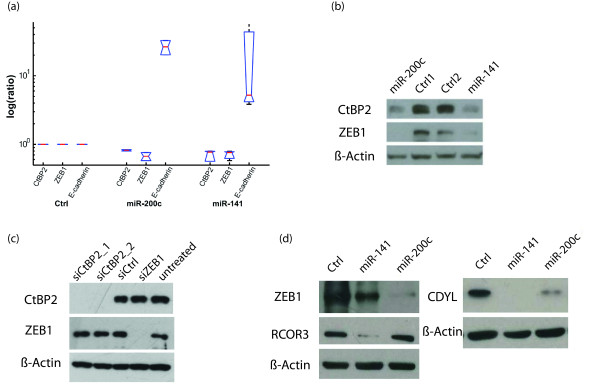
**Protein complexes regulated by the *miR-141-200c *cluster**. **a**, Real-time reverse transcription-PCR of *CtBP2 *and *ZEB1 *after transfection of the indicated miRNAs in undifferentiated cancer cells (PANC-1). The expression levels of *E-cadherin *(of which the transcription is repressed by *CtBP/ZEB complex*) are included as positive controls. **b**, Confirmation of the regulation of CtBP2 and ZEB1 by miR-141 and miR-200c on protein levels by immunoblots. **c**, ZEB1 and CtBP2 knock down by siRNAs, no change in protein levels of the respective complex partner is oberserved. e, Downregulation of CDYL and RCOR3 on protein level when miR-141 or miR-200c was transiently transfected.

We showed that overexpression of *miR-141 *and *miR-200c *led to reduced expression of *CtBP2 *and *ZEB1 *in human pancreatic carcinoma (PANC-1) cells (Figure 4a). Luciferase reporter assay showed reduced activity of the CtBP2 and ZEB1 3'UTR-luciferase reporters with increased levels of miR-141 and miR-200c (Additional file 8, Figure S1 online). These results are also confirmed on protein level by immunoblots (Figure 4b). In order to rule out the possibility that the stability of ZEB1 and CtBP2 are dependent on each other, we separately knocked down ZEB1 and CtBP2 by siRNAs in PANC-1 cells and observed no change in protein levels of the respective complex partner (Figure 4c). Although the expression of CDYL and RCOR3 is less obviously affected by overexpression of miR-141 and miR-200c in PANC-1 cells as compared to CtBP2 and ZEB1 (data not shown), we observed a downregulation of CDYL and RCOR3 on the protein level, when miR-141 or miR-200c were transiently transfected in PANC-1 cells (Figure 4d), suggesting that CDYL and RCOR3 are also targets of the miR141-200c cluster. Together, these experiments demonstrate, for the first time, that CtBP2, CDYL and RCOR3 can be regulated by miR141-200c cluster post-transcriptionally. As the functional consequence of miRNA overexpression, the expression of *E-cadherin *mRNA is greatly upregulated (Figure 4a), indicating that the repression activity of *CtBP/ZEB *complex is compromised. The interaction between the *miR-141-200c *cluster and multiple components of the *CtBP/ZEB *complex suggests a coordinated regulation of the repression activity for the *CtBP/ZEB *complex. Intriguingly, the *miR-141-200c *cluster also targets *β-catenin*, which is a shared component of cell adhesion and *Wnt *signalling [38]. β-catenin is found in the plasma membrane, where it promotes cell adhesion by binding to E-cadherin, in the cytoplasm, where it is easily phosphorylated and degraded in the absence of a Wnt signal, and in the nucleus, where it binds to TCF transcription factors and induces the transcription of Wnt target genes. Most protein-interacting motifs of β-catenin overlap in such a way that its interactions with each of its protein partners are mutually exclusive [38]. Since the miR-141-200c cluster and E-cadherin are both downregulated during EMT, it is tempting to speculate that more β-catenin would be made available for participating in transactivating downstream genes, which may contribute to the progress of cancer [4].

## Discussion

MicroRNAs and their functions have been a fascinating research topic in recent years [8,39,40]. In animals, miRNA-guided regulations of gene expression are likely to involve hundreds of miRNAs and their targets. Genetic studies have successfully elucidated some miRNA activities, termed genetic switches, which have intrinsic phenotypic consequences [8,40]. miRNA activities can be classified based on whether their major effect is conveyed through one, a few or many targets (from tens to hundreds). All genetic switches discovered so far belong to the former class (a few targets). It is unclear how the latter class, termed target battery [8], which might be subtly regulated on the protein level [6,7], contributes to proper phenotypes.

In this study, we completed a comprehensive analysis of human protein complexes, which might be co-ordinately regulated by miRNAs. When this paper was under review, Tsang *et al*. [12] predicted human microRNA functions by miRBridge to assess the statistical enrichment of microRNA-targeting signatures in annotated gene sets, including our CORUM protein complexes [20]. These protein complexes can be considered as examples of "target battery" [8]. Our statistical analysis suggests that, by simultaneously targeting several components of protein complexes, a single miRNA or co-expressed miRNAs may have cumulative effects. To demonstrate this, we experimentally verified that the miR141-200c cluster interacts with four different components of the CtBP/ZEB complex. Interestingly, although Tsang et al. used their own miRNA target predition, which is different from TargetScan prediction, their protein complex result also included the interaction of the miR200 family and CtBP complex [12] which includes miR-200c. This supports our finding that the miR141-200c cluster also interacts with the CtBP complex. The functional analysis of the miRNA-regulated protein complexes revealed a clear bias towards transcriptional regulation, signal transduction, cell cycle and chromatin regulation, for which confirmation has been reported only by individual experimental studies of selected miRNAs. Our approach provides improved candidate miRNA target lists to the experimentalist, as demonstrated by a benchmark against large-scale, quantitative proteomics data.

Some ancient miRNA genes are deeply conserved in the kingdom Animalia [37,38] or in the kingdom Plantae [41] while during the evolution, novel miRNA genes were constantly created, fixed or lost [42-45]. Interestingly, the genomic organization of some miRNA clusters were well preserved for millions of years, implying a functional incentive to keep such configurations [5,46]. The evolution of homogeneous miRNA clusters can be easily explained by the classical gene duplication theory [47]. The regulatory effect of such clusters might merely be an increase of dosage. The evolution of hetergeneous miRNA clusters is more complicated. Two different miRNAs can be located near each other by various genomic events, such as recombination, transposon insertion, etc. Or large number hairpin repeats might evolve into miRNAs of different families. For example, the largest human miRNA cluster miR-379-656 [46] consists of different miRNA families, which evolved by tandem duplication of an ancient hairpin sequence. Once a newly formed miRNA cluster proves to provide a functional advantage, which might be co-ordinate regulation of protein complexes, the genomic organization of such a cluster could be fixed by evolution [43].

In eukaryotic cells, RNA operons, mostly sequence-specific RNA binding proteins, may co-ordinately regulate functionally related mRNAs to aid the formation of macromolecular protein complexes [48]. In such a scenario, mRNAs of different components of a protein complex are brought together by associating with specific RNA operons. The localization of these mRNAs might also facilitate the simultaneous interaction of miRNAs and their corresponding target mRNAs. Interestingly, RNA operons bind to motifs, which are sometimes located in the 3'UTRs of mRNAs. Thus, the competition or cooperation between miRNA binding and RNA operon binding might be a research topic worth pursuing.

## Conclusion

The results presented here can be used as a starting point for experimentalists to systematically evaluate miRNAs and targets interactions at a systems level. The concept that coexpressed small RNAs may synergistically target protein complexes for a more efficient regulation is of course not limited to animal miRNAs.

## Authors' contributions

SS and SD designed the statistical analyses, interpreted the results. UB, SSB, YW and TB designed miR141-200c related experiments. UB and SSB performed experiments. UB, SSB, YW and TB interpreted the results. DL, AK, KFM, and AR contributed to data analysis. YW conceived the idea. YW and FT coordinated the study, interpreted the results. SS, SD and YW wrote the manuscript. All authors have read and approved the manuscript.

## Supplementary Material

Additional file 1 Supplementary Table S1, complexes that are significantly targeted by single miRNAs (P-value < 0.05, Fisher's exact test) Click here for file

Additional file 2 Supplementary Table S2, complexes that are significantly targeted by clusters of miRNAs (P-value < 0.05, Fisher's exact test) Click here for file

Additional file 3 Supplementary Table S3, over- and underrepresentation of Gene Ontology terms in complex members that are targeted by miRNAs Click here for file

Additional file 4 Supplementary Table S4, over- and underrepresentation of KEGG pathways in complex members that are targeted by miRNAs Click here for file

Additional file 5 Supplementary Table S5, miRNA target sites inferred by PAR-CLIP in experimentally verified protein complexes Click here for file

Additional file 6 Supplementary Table S6, average Pearson correlation of miRNAs that simultaneously target a specific complex Click here for file

Additional file 7 Supplementary Table S7, target interconnectivity of miRNA clusters Click here for file

Additional file 8 Supplementary Figure S1, reduced activity of the CtBP2 and ZEB1 3'UTR-luciferase reporters with increased levels of miR-141 and miR-200c Click here for file
