# Supplementary figures and images for: MicroRNAs coordinately regulate protein complexes
Source: BMC Syst Biol. 2011 Aug 25;5:136. doi: 10.1186/1752-0509-5-136 (PMC3170341; doi:10.1186/1752-0509-5-136)

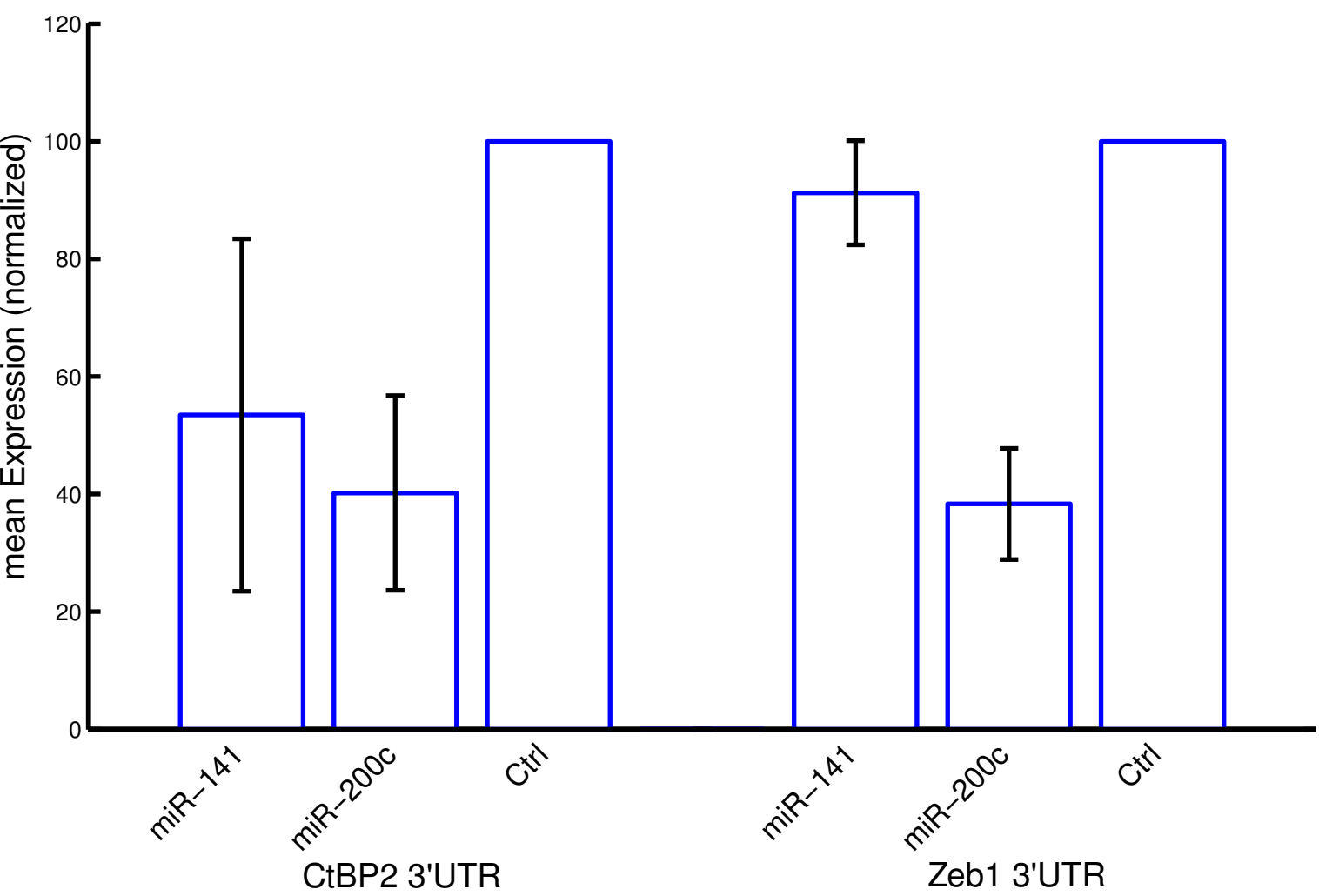

Supplement: Additional file 8 — Supplementary Figure S1, reduced activity of the CtBP2 and ZEB1 3'UTR-luciferase reporters with increased levels of miR-141 and miR-200c [file 1752-0509-5-136-S8.PDF]
